# Necrotizing Sialometaplasia of Palate: A Case Report

**DOI:** 10.1155/2012/679325

**Published:** 2012-08-22

**Authors:** N. G. Garcia, D. T. Oliveira, S. E. S. Faustino, A. L. R. Azevedo

**Affiliations:** ^1^Department of Stomatology, Area of Pathology, Bauru School of Dentistry, University of São Paulo, 17012-901 Bauru, SP, Brazil; ^2^Dentist, Private Practice, Brasília, Federal District, Brazil

## Abstract

*Background*. Necrotizing sialometaplasia (NS) is an uncommon benign reactive necrotizing inflammatory process involving minor salivary gland that often mimics malignancy both clinically and histopathologically. *Case Report*. We report the case of a healthy 26-year-old man with a painless swelling in the hard palate near the middle raphe, asymptomatic, well limited, and raised edges. The patient was submitted to incisional biopsy and histopathological examination. The histological diagnosis was necrotizing sialometaplasia. *Discussion*. The clinical and histological similarity between this entity and a malignant lesion implies a risk of unnecessary or mistreatment. Therefore, clinicians and pathologists should be aware of this lesion as to avoid errors in the diagnosis and treatment of this benign pathologic condition.

## 1. Background

Necrotizing sialometaplasia (NS) was first reported in 1973 by Abrams et al. as reactive necrotizing inflammatory process involving minor salivary gland of hard palate [1]. In the WHO classification of salivary gland tumors, NS is classified under the group of tumor-like lesions [2]. These lesions are relatively rare and benign. However, may be confused with malignant lesions both histopathologically and clinically, particularly squamous cell carcinoma and mucoepidermoid carcinoma [3, 4]. We report the clinical and histopathological features of a case of necrotizing sialometaplaia of the hard palate in a young adult man.

## 2. Case Report

**Figure 1:**
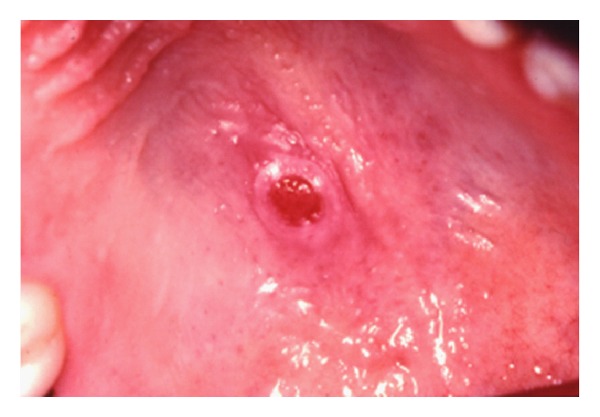
The clinical photograph shows a lesion on the posterior hard palate, asymptomatic, well limited, and raised edges.

A 26-year-old man presented with a painless swelling in the hard palate. He was referred to dentist that noted in a clinical examination a lesion with 1.5 × 0.5 cm on the posterior aspect of the hard palate near the middle raphe, asymptomatic, well limited, and with raised edges (Figure 1). Thus, an excisional biopsy was performed under local anesthesia and the specimen was submitted to the Oral Pathology Biopsy Service of Bauru School of Dentistry, University of São Paulo.

**Figure 2:**
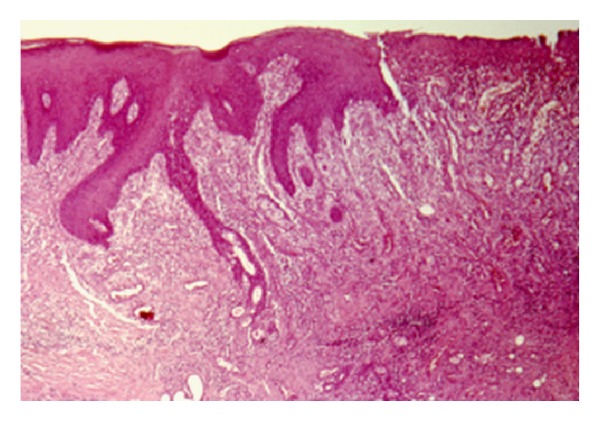
Histopathological picture shows a reactive necrotizing inflammatory process involving the minor salivary glands, with pseudoepitheliomatous hyperplasia of the overlying epithelium, but without evidence of dysplasia.

**Figure 3:**
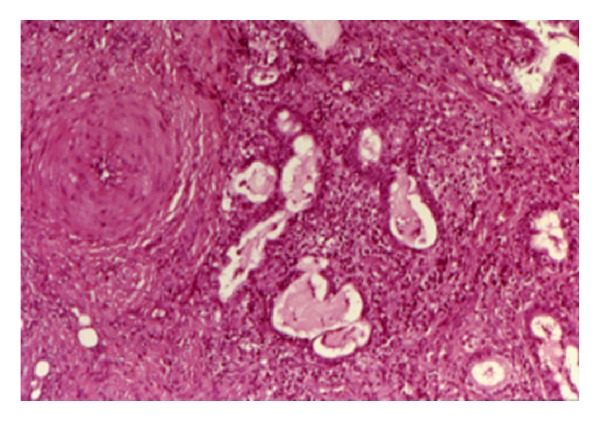
In the fibrous connective tissue were found, squamous metaplasia of the salivary ducts and acinar necrosis with preservation of the lobular architecture of the neighbouring minor salivary glands.

**Figure 4:**
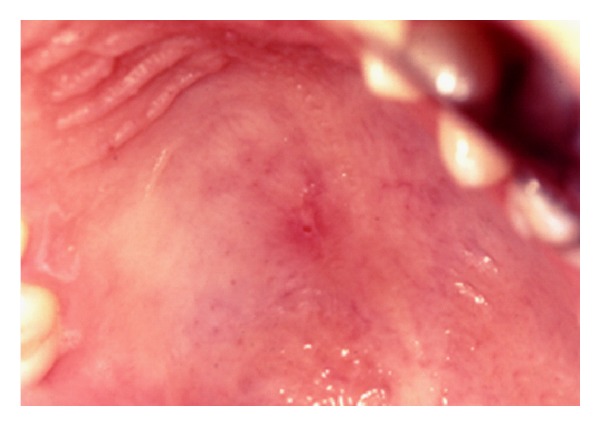
After one month the lesion regressed.

The histopathological examination revealed a reactive necrotizing inflammatory process involving the minor salivary glands, with pseudoepitheliomatous hyperplasia of the overlying epithelium, but without evidence of dysplasia (Figure 2). In the fibrous connective tissue, squamous metaplasia of the salivary ducts, and acinar necrosis with preservation of the lobular architecture of the neighbouring minor salivary glands were found. Chronic inflammatory cells surrounding the glandular tissue were also observed (Figure 3). After one month the lesion regressed (Figure 4).

## 3. Discussion

Since necrotizing sialometaplasia was first described in 1973, by Abrams et al., about 200 cases were reported [1]. Today, it is known that NS arises since in childhood to old age. This lesion is a benign and self-limiting inflammatory condition which can be found at any site in the body that contains elements of salivary gland, from the paranasal sinuses to the lung, but most of the cases have still been reported in the oral cavity [5].

Although the exact etiology of NS is not fully resolved, the lesion is believed to be related to a physicochemical or biological injury on the blood vessels that would produce ischemic changes, leading to infarction of the salivary gland acini with posterior necrosis, inflammation and intent of repairing, inducing metaplasia, changes in ducts, and further cicatrization [6]. Usually a local trauma like those produced by intubation, local anesthesia, surgery procedures, use of unadapted dental prothesis, violent or induced vomiting like in patients with bulimia, infectious processes, radiotherapy, and use of tobacco or cocaine, due to its constrictor effect, could be the factors involved in local ischemia [7].

The ordinary lesion presents itself as a deep-seated palatal ulcer with clinical and histologic findings mimicking those of a malignant neoplasm. Unfortunately, the NS is still easily misdiagnosed as a mucoepidermoid carcinoma or a squamous cell carcinoma. In such cases, the microscopic examination is mandatory, because failure to recognize NS may result in unnecessary radical surgery with undesirable mutilating effects.

According to Anneroth and Hansen [8], the pathogenesis of NS can be divided into five histologic stages: infarction, sequestration, ulceration, the reparative stage, and healed stage. Moreover, according to Imbery and Edwards, squamous metaplasia of the salivary ducts and coagulation necrosis of the acini is observed in early lesions and reactive fibrosis during the late stage of NS. [6]. Necrosis of the glandular acini predominates in the infarct stage. An extensive infarct leads to sequestration of the necrotic acini, resulting in ulceration. Pseudoepitheliomatous hyperplasia could develop during the healing process of ulceration. However, if the infarct is limited in its extent, then sequestration and ulceration do not occur, and necrosis might occur just in a small portion [6].

The repair of ductal epithelium and acini by squamous metaplasia with accompanying pseudoepitheliomatous hyperplasia can actually be mistaken microscopically with mucoepidermoid carcinoma or squamous cell carcinoma [6]. It should be emphasized that these lesions also affect adults and children as well as in NS [2].

Most of reported cases of NS have emphasized that an incisional biopsy must be performed on these lesions and careful histopathologic examination should be done to determine the proper treatment and management regimen [9]. However, recently, Lee et al. reported a case of necrotizing sialometaplasia and adenoid cystic carcinoma coexisting simultaneously and they reinforce that the result of incisional biopsy might be different depending on the exact site where the biopsy is taken. They also discussed two possible explanations for the pathogenesis of NS. First, the incisional biopsy or the local anesthesia that was administered for the biopsy could be a possible cause of the necrotizing sialometaplasia. Second, the pressure from the tumor on the adjacent vascular structures could have helped generate the necrotizing sialometaplasia [9].

In conclusion, clinicians and pathologists should be aware of this lesion as to avoid errors in the diagnosis and treatment of this benign pathologic condition.
